# Covered smut screening in barley: power analysis and effect on agronomic traits

**DOI:** 10.1186/s13007-026-01577-8

**Published:** 2026-07-27

**Authors:** Gopika Gopinathan, Fluturë Novakazi, Ines Berro, Julie Dawson, Therése Bengtsson, Lucía Gutiérrez

**Affiliations:** 1 Department of Bacteriology, University of WI–Madison, 1550 Linden Dr., Madison, WI 53706 USA; 2 Department of Crop Health, Faculty of Agriculture, Civil and Environmental Engineering, University of Rostock, 18059 Rostock, Germany; 3 Department of Biometrics and Statistics, Agronomy College, Universidad de la República, Av. E. Garzón 780, 12900 Montevideo, Uruguay; 4 Department of Plant and Agroecosystem Sciences, University of WI–Madison, 1575 Linden Dr., Madison, WI 53706 USA; 5 Department of Plant Breeding, Swedish University of Agricultural Sciences, Sundsvägen 10, 23456 Alnarp, Sweden

**Keywords:** Barley, Covered smut, Germination, Protocol, Speed breeding, Power analysis

## Abstract

**Background:**

Covered smut in barley caused by *Ustilago hordei* leads to yield reduction and quality loss of stored grains and is especially challenging in organic production. However, screening for resistance remains challenging. The goal of our research was to evaluate protocols for screening covered smut in barley under normal and speed breeding conditions that could be scaled up for breeding purposes. We considered favorable pathogen growth conditions, a sufficient sample size to detect differences among genotypes through a power analysis, sources of disease escape or avoidance, and the infection effect on agronomic traits.

**Results:**

In the first experiment, twenty genotypes treated with various inoculum concentrations were screened for disease incidence under a speed breeding system. Generally, low infection levels were found, likely due to disease escape or avoidance. Based on a power analysis, we modified the protocol to include more plants and improved pathogen growth conditions under a normal greenhouse system. With the modified protocol, the incidence of covered smut was significantly different among genotypes. The protocol also reduced the number of plants required to detect at least one infected plant. Artificial inoculation significantly decreased germination rates while head emergence, days to heading, and plant height were affected by disease infection in the most susceptible genotypes. We also found that covered smut incidence varied with tiller emergence order. The genotypes ‘DH160779’ (RES check), PI 270630’, ‘CIho15270’, and ‘MTV-color-158’ presented potential resistance to covered smut.

**Conclusion:**

The protocol has a high power to differentiate moderately resistant barley genotypes and we confirmed that specific agronomic traits were affected by disease incidence in susceptible genotypes.

**Supplementary Information:**

The online version contains supplementary material available at 10.1186/s13007-026-01577-8.

## Introduction

### Covered smut infection in barley and its importance

Organic grain production is strongly constrained by spike diseases such as Fusarium head blight, bunts, smuts, and ergots [1–3]. Several smut diseases in cereals are caused by fungi from the *Ustilago* genus, including loose smut in barley (*U. nuda*) [4], and wheat (*U. tritici*) [5] and covered smut in oats (*U. avenae*) [6] and barley (*U. hordei*) [7]. Covered smut in barley caused by *U. hordei* (Pers.) Lagerh is a seedborne and partially soilborne basidiomycete fungus that infects seedlings soon after germination [7–9]. It has a predominantly sexual reproductive cycle [10, 11]. Teliospores from infected spikes contaminate healthy seeds at harvest and remain dormant until the next season [7, 10, 12]. Following planting, the teliospores germinate on the surface of the seed to produce promycelium with basidiospores, which fuse to form the dikaryotic mycelium responsible for host infection [13, 14]. The dikaryotic hyphal stage penetrates directly into the coleoptile during seedling emergence and expands its hyphae within the seedling [13]. When infected by covered smut, highly resistant barley cultivars exhibit defense responses such as callose deposition at the penetration site (coleoptile), which restricts further fungal penetration and, in some cases, could result in termination of fungal growth [11, 15]. However, in compatible interactions, such as those occurring in susceptible genotypes, *U. hordei* survives through the vegetative phase and infects the apical meristem [16]. In infected plants*,* kernels are replaced by black smut sori, and the parts of the spike converted to sori are covered by a silvery membrane [7, 8, 17]. During severe infection, tips of upper leaves can show pustules, smutted culms, and streaks of smut that extend to the leaf sheath and nodes [8]. Hence, smut symptoms are observed only as the plant approaches flowering [18].

Covered smut in barley was a major constraint in North American barley production in the early to mid-1900s [19, 20]. Between 1917 and 1921, covered smut caused an estimated loss of 3.5 million bushels of barley in the United States, representing ~ 2% of the national production [21]. The economic loss corresponded to approximately USD 3.5 million in 1921 (equivalent to approximately USD 65 million in 2026 USD, adjusted for inflation using the U.S. Consumer Price Index) [22]. Covered smut also leads to a reduction in seed quality during storage due to the presence of black smut balls among the seeds [23, 24]. Barley grains contaminated with covered smut balls are unacceptable for food, malting purposes, or commercialization [24–26]. In the U.S., barley grains for marketing are considered as “smutty grade” if more than 0.2% of the grains are converted to smut balls [27]. However, several state seed certification rules restrict the certification of barley seeds with or above 0.5–2% smutted seeds per plot [28, 29]. The advancement of fungicidal seed treatment methods in the twentieth century greatly reduced the incidence of covered smut in barley [30–33].

### Methods to control and screen covered smut in barley

Covered smut can be controlled by physical, biological, and chemical seed treatment methods, as well as the use of disease-resistant cultivars. While covered smut is largely controlled by fungicide seed treatments in conventional production, fungicide application restrictions under organic cropping systems pose a challenge for disease control in organic barley production [34, 35]. Even in conventional systems, seed fungicide usage has been discouraged because of its adverse effects on human health, such as the risk of endocrine disruption and congenital disorders [36, 37]. Additionally, some evidence shows that *U. hordei* has potentially developed genetic fungicide resistance in conventional systems because of the repeated application of fungicides with the same mode of action [38–40]. In the past, simple and cost-effective treatments, such as formalin disinfection and hot-water treatment, were commonly used to prevent the transmission of covered smut infection in barley [19, 41, 42]. Steam treatment has attracted increasing interest as an effective method for controlling smut in organic systems [43]. Smut and bunt infections in organic production have also been managed with wheat flour and biocontrol agents [44–46]. 

Given the challenges in controlling covered smut in barley in both organic and conventional systems, one of the most effective solutions is to develop resistant cultivars [24, 25, 47]. Covered smut resistance in barley is controlled by multiple genes of major effects [25, 48, 49], but evidence of positive transgressive segregation supports an additional quantitative genetic basis [50, 51]. Other studies have explained the variation in disease expression among barley genotypes as a gene-for-gene resistance mechanism [52], where specific host resistance genes recognize and restrict infection by corresponding races of the covered smut pathogen [20]. So far, seven distinct resistance genes against barley covered smut have been identified but only three, have been mapped [48, 49, 52–54]. Therefore, resistance to covered smut in barley could be quantitative, qualitative, or both. Several covered smut resistance sources have been identified in barley with moderate to high levels of resistance [48, 50, 55]. Understanding the mechanism of resistance and development of covered smut-resistant cultivars requires protocols to efficiently inoculate plants and screen for disease [8, 56–60].

### Limitations of current screening approaches

Screening for covered smut resistance presents several challenges. Disease escape or avoidance is common, even in susceptible genotypes, meaning that not all inoculated plants develop visible symptoms [25, 60–63]. This complicates the differentiation between susceptible and moderately resistant genotypes and can lead to underestimation of disease incidence.

Several factors contribute to this limitation. First, the infection process of *U. hordei* is relatively slow compared to host development, which can result in plants escaping infection if they reach reproductive stages before the pathogen fully colonizes the apical meristem [64]. For example, plants may contain fungal mycelia at early growth stages but fail to develop symptoms at heading [60].

Second, a potential limitation is the effect of infection in seed germination. If infected seeds fail to germinate, disease incidence may be underestimated because infected plants are not observable. Although this effect has not been extensively documented for covered smut, similar reductions in germination have been reported for other smut diseases [61, 65] through damage to their plumule or radicle [66, 67].

Third, suboptimal environmental conditions—such as temperature, moisture, and soil composition—can limit infection efficiency [8, 62, 68, 69]. Cool temperatures (10–22 °C), acidic soil pH (5–6), and soil moisture are known factors to affect infection [8, 62, 68]. Additionally, soil structure and inoculum distribution influences the likelihood of successful pathogen establishment [68].

Fourth, inadequate sample sizes reduce the statistical power to detect infection, particularly when disease incidence is low [63]. This is especially problematic when attempting to distinguish intermediate resistance levels, which may require larger populations to detect meaningful differences [50, 51].

Finally, screening is constrained by the late expression of symptoms [7, 8, 25]. Because covered smut symptoms appear only at heading, there has been interest in accelerating plant development using speed breeding systems to reduce evaluation time [70–72]. However, faster development time may also increase the risk of disease escape.

In essence, adequate plant numbers and well-designed experiments are essential for reliable disease symptom observation and characterization of disease genetic architecture [73, 74]. Additionally, there is a two-way interaction of covered smut with agronomic traits that could shape disease avoidance. In crops such as wheat, dwarf bunt causes a reduction in plant height [75] and delayed flowering [76]. Delayed flowering has also been reported with pearl millet smut [77]. In barley covered smut, Mathre [78] reported that infection affects the emergence of the head out of the leaf sheath, days to heading, and plant height. Similarly, Nohtani et al. [79] reported that plant height is significantly reduced by covered smut infection. Understanding these relationships is key for selecting disease resistant genotypes [79, 80]. Ideally, the inoculation protocol should consider all the above-mentioned factors.

### Objective of this study

The goal of this research was to evaluate and optimize the protocols for screening covered smut in barley under normal and speed breeding conditions, estimating the sample size to detect differences among genotypes through a power analysis, evaluating sources of disease escape or avoidance, and to evaluate the effect of disease incidence on agronomic traits. Specifically, our goal was to design a protocol to screen for covered smut resistance in barley by accelerating the growth cycle via a speed breeding system for faster detection of the disease. Since this did not allow us to detect genotypic differences, we performed a power analysis and modified the protocol to fit a normal greenhouse system with more favorable pathogen conditions. Furthermore, we studied the effects of covered smut on seed germination, head emergence, days to heading, and plant height. Finally, to assess the growth pattern of the fungus in normal and speed breeding systems, we allowed most of the barley tillers to develop by providing nutrients and water for a prolonged period, possibly increasing the likelihood of the extended presence of fungal mycelia near the meristematic tissues.

## Materials and methods

The protocol evaluation strategy was accomplished via artificial inoculation and evaluation of the incidence of covered smut in the main head and its effect on agronomic traits under different growth systems (i.e., speed breeding (*speed main head* experiment) and normal greenhouse (*normal main head* experiment)). The effects of inoculation on all tillers were also studied in the two systems: speed breeding (s*peed tillering* experiment) and normal greenhouse (*normal tillering* experiment). Finally, a germination experiment was conducted to understand the effect of the pathogen on seed germination.

### Plant material

Twenty spring barley genotypes from breeding programs in the U.S. Midwest Region, Pacific Northwest Region and Canada were studied for covered smut resistance. No region-specific differential lines have been developed for our region;: consequently, one putatively resistant genotype, ‘DH160779’ (RES check), and one potentially susceptible genotype, ‘DH160799’ (SUS check), were included in the experiments. The designation of these checks was based on unpublished observations from collaborator studies (B. Meints, personal communication), but do not represent established standatds. For the *speed main head* experiment, the full set of 20 genotypes was used, which consisted of 15 breeding lines, two advanced breeding lines (‘Purple Prince’ and ‘White Queen’), and three cultivars (‘CDC Clear’, ‘Full Pint’ and ‘Quest’) (Additional file 1: Table S1), whereas a subset of eight genotypes selected on the basis of potential resistance and potential susceptibility in the *speed main head* experiment was used for the *normal main head* experiment. The *speed tillering* experiment evaluated two genotypes, whereas eight genotypes were evaluated in the *normal tillering* experiment. Eight genotypes were also evaluated in the germination test.

### Preparation of the inoculum

Smut heads (hard smut balls) from barley regional trials conducted at the West Madison Agricultural Research Station (43° 03′ 37″ N, 89° 31′ 54″ W) were used. Samples consisted of an inoculum mixture of smut heads from the 2021, 2022, and 2023 growing seasons, collected either directly from recently harvested grain (i.e. season 2023), or barley samples stored in the cold-room at the Cereals Breeding and Quantitative Genetics Laboratory from the 2021 and 2022 growing seasons. The regional barley trials conducted at West Madison Agricultural Research Station, used seed sources sent from the department of Crop and Soil Science, Oregon State University (44° 34′ 00″ N, 123° 17′ 07″ W) which likely contained covered smut spores. Because no molecular characterization or sequencing was conducted to identify the specific races of *U. hordei*, the inoculum used in this study is assumed to represent a mixture of races commonly present in U.S Midwest and Pacific Northwest fields. The smut balls containing numerous teliospores were collected and preserved in paper bags in a cold chamber at 4 °C.

We adapted the method of Tapke [56] for inoculum preparation. For the preparation of the teliospore smut powder, the smut balls were crushed with a mortar and pestle. A fine sieve (150 µm) and a soft brush were used to transfer the smut powder into an airtight glass container. Teliospore germination and promycelium formation were observed under a microscope as described in Additional file 1: Table S2; however, spore counting was not conducted. For speed breeding experiments, a fungal spore suspension was prepared by adding 1 L (L) of distilled water to 1 g or 2 g of prepared smut powder in separate glass bottles to obtain inoculum levels of 1 g L^−1^ and 2 g L^−1^, respectively. The glass bottles were tightly closed and placed on an orbital shaker for homogenization of the inoculum spore suspensions. Inoculum spore suspensions were used immediately after preparation. For the normal experiments and the germination test, new batches of inoculum suspensions were freshly prepared by adding infected heads from previous speed breeding experiments to the available smut powder mix. The inoculum suspension was stored at 4 °C after seed treatment for the double inoculation procedure.

### Artificial inoculation via seed treatment

Three racks with 500 mL cone tubes were arranged for the non-inoculated control, 1 g L^−1^, and 2 g L^−1^ seed treatments. For each genotype, 10 g of seeds were used for each treatment. For artificial inoculation, 50 mL of spore suspension from 1 and 2 g L^−1^ glass bottles were added to the cones on the respective racks. The non-inoculated control was left untreated. The racks with the inoculated tubes were placed on an orbital shaker for 2–3 min to ensure that the inoculum was well distributed on the surface of the seeds. After removing the tubes from the orbital shaker, the seeds were immersed in the inoculum suspension for an additional 20 min. The suspension was then decanted. The seeds were allowed to dry in the dark at 15 °C on dry paper towels for two days. The non-inoculated control, 1 g L^−1^, and 2 g L^−1^ were used for the speed breeding experiments, whereas the 1 g L^−1^ inoculum was used for both the normal experiments and the germination experiments. The experiments and germination tests were conducted immediately after the seeds were dried. To check teliospore germination in the seeds, 5 seeds per pot of the genotype SUS check were planted at 1 g L^−1^ and 2 g L^−1^. The seeds were removed 24 h after planting, and teliospore germination was checked (Additional file 1: Table S2).

Owing to the possibility of teliospores washing away from the seed surface during watering, we used a double inoculation method in the normal experiments to promote uniform teliospore spread within the growing media. In the first inoculation, the seed was inoculated with the spore suspension as described above, while the second inoculation consisted of applying 0.5 mL of spore suspension (1 g L^−1^) to each pot prior to seedling emergence.

### Experimental design

For the *speed main head* experiment, we used an alpha incomplete block design with a factorial arrangement of 20 genotypes at three inoculum levels (non-inoculated control, 1 g L^−1^, and 2 g L^−1^), with three replications and eight plants per experimental unit. Individual plants grown in separate 0.8-L plastic pots (3.1 cm radius × 25.4 cm height) were treated as subsamples within experimental units. We used a randomized complete block design with three replications and 78 plants per experimental unit in the *normal main head* experiment. Each plant was in a 0.3 L plastic pot (2 cm radius × 21 cm height) and was considered a subsample within the experimental unit. Both the *speed tillering* and *normal tillering* experiments were conducted using a randomized complete block design, with six replications for the *speed tillering* experiment and five for the *normal tillering* experiment without subsampling. Each experimental unit was a 7.6 L plastic pot (10.8 cm radius × 20.9 cm height). Finally, for the germination test, we used an alpha incomplete block design with a factorial arrangement of treatments of eight genotypes and two inoculum levels (non-inoculated control and 1 g L^−1^) with three replications where each replication had 100 seeds in a single roll towel which was considered an experimental unit.

### Growing conditions and planting

The experiments were conducted in the Walnut Street greenhouse facilities at the University of Wisconsin–Madison (43° 04′ 34″ N, 89° 25′ 26″ W). During the experimental period, the light intensity was maintained between 450 and 500 μmol m^−2^ s^−1^. TotalGrow high-intensity top-light 330 W white lamps (TotalGrow Lights, Holland, MI, USA) were used.

For the speed breeding experiments, the *speed main head* and *speed tillering*, controlled conditions in the greenhouse with 22 h of light at 22 °C, with lights on from 5 to 3 AM, were used in combination with 2 h of darkness at 16 °C [70]. Planting was conducted for the *speed main head* and *speed tillering* experiments on February 27, 2024, and March 13, 2024. PRO-MIX® (pH = 5.8–6.2) (Premier Tech Growers & Consumers, Rivière-du-Loup, Québec, Canada) was used as the planting medium.

**Fig. 1:**
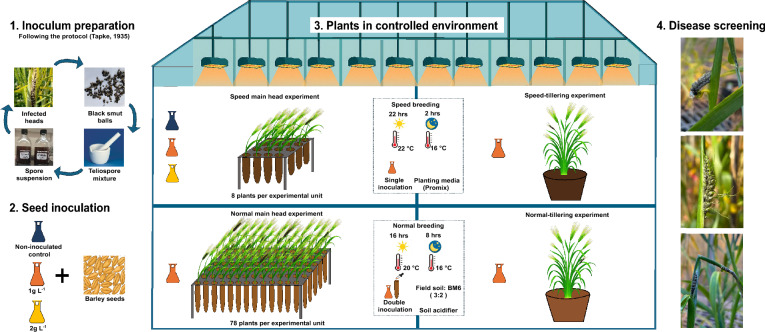
Protocols used to inoculate covered smut in barley and screen for disease under normal and speed breeding systems. The steps for the protocol were as follows: **1** inoculum preparation, **2** seed inoculation, **3** plant growth in a controlled environment, and **4** disease screening. Details can be found in the text

For the normal greenhouse experiments, the *normal main head and normal tillering,* controlled conditions in the greenhouse with 16 h of light at 20 °C with lights on from 5 AM to 9 PM, were used in combination with 8 h of darkness at 16 °C (Fig. 1). Planting was conducted for *normal main head* and *normal tillering* experiments on October 15, 2024, and October 16, 2024, respectively. A mixture of sterilized field soil available at the Walnut Street greenhouse facilities and BM6 (pH = 5.4–6.2) (Saint-Modeste, Quebec, Canada) at a ratio of 3:2 (v/v) was used as the planting medium. Because the soil structure influences covered smut growth [8, 69], we used a mixture of pot mixture BM6 and field soil as growth media for the normal greenhouse experiments.

All the experiments, including *speed main head*, *speed tillering*, *normal main head* and *normal tillering*, were fertilized with ∼30 g of Burpee organic all-purpose plant food (Warminster, Pennsylvania, USA) to the surface of the pots at the four-leaf stage (ZGS 14, [81]). Additionally, for the *speed tillering* and *normal tillering* experiments, a second round of fertilizer application was conducted at the booting stage of the main tiller to increase the growth period of the plants. The macronutrient proportions present in the plant food were 5% nitrogen, 5% phosphate, and 5% potash, along with micronutrients such as 0.5% magnesium, 1% sulfur, 0.1% iron, 0.05% manganese derived from poultry litter, feather meal, the bone meal and sulfate of potash and a natural biomix blend of beneficial microbes (1.51%; Bacillus, Rhizophagus, Septoglomus, and Claroideoglomus). The plants were watered daily, and stakes were used to avoid lodging. A mixture of SuffOil-X (BioWorks Inc., Victor, New York, USA) and castor oil was applied to the foliage to prevent infection caused by powdery mildew (*Blumeria graminis*). Yellow sticky traps were used to control aphids and other insect pests. In the *normal main head* and *normal tillering* experiments, acidic pH was maintained via an Espoma organic soil acidifier (Millville, New Jersey, USA) with 30% sulfur by applying a layer to the surface of the pots one day after planting, as *U. hordei* has been reported to show relatively high infection rates at pH 5–6 [8].

### Phenological evaluation and disease scoring

Zadok’s growth scale (ZGS, [81]) was used to score the phenological stages. In the *normal main head* experiment, the appearance of the first leaf (ZGS 11) was scored as seed emergence (yes/no), which was used to calculate the percentage of seeds that did not emerge. In the *speed main head* and *normal main head* experiments, measurements were taken on the main head. The date when the main head emerged (ZGS 59) was scored as days to heading. The plant height at ZGS 59, measured from the soil surface to the tip of the head (excluding awns), was scored as the plant height. Disease incidence (DI) was scored as a ‘yes’ when the main head was converted to black smut sori (DI = 1) or ‘no’ when no black smut sori was detected (DI = 0). For the *normal main head*, days to heading were only scored if the head emerged smoothly out of the boot, i.e., head emergence = (yes). In the *speed tillering* and *normal tillering* experiments, measurements were taken from the heads of several tillers. In the *speed tillering* experiment, the days to heading and covered smut incidence were scored for the head of each tiller. In the *normal tillering* experiment, head emergence, days to heading, and covered smut incidence were scored on the heads of the first seven tillers.

### Germination test

The roll towel method adapted from USDA ARS [82], was used to conduct germination tests via Avantor 8002 grade seed testing paper (Radnor, Pennsylvania, USA). A seed testing sheet was moistened with distilled water and placed on a disinfected surface. Seeds (i.e., non-inoculated or inoculated with 1 g L^−1^) of a genotype were placed in two rows horizontally near the top edge of the paper. Another moistened seed testing paper was placed on top and rolled together to form a cylindrical paper roll. The paper rolls were placed upright in a container filled with distilled water to submerge the bottom third. The container was kept at 25 °C for 5 days. After 5 days, the paper towels were unrolled, and the number of seeds showing radicle emergence (ZGS 05) were considered germinated and counted, which was used to calculate the percentage of seeds that did not germinate.

### Power analysis

A power analysis was conducted on the basis of the results obtained in the *speed main head* experiment to determine the number of plants needed for the next experiment (*normal main head*). The cumulative distribution function of the binomial distribution was used to estimate the power. Assuming that the number of infected plants (*X*) follows a binomial distribution (*X* ⁓ Bin(n,*p*)), P(*X* ≥ 1| p_0_) ≤ α, which makes Power = P(*X* ≥ 1| p_1_), and therefore Power = 1—P(*X* ≤ 1| p_1_). Therefore, for the simplified case of detecting at least one infected plant, a power function was built with the following equation for a range of plants evaluated (n):

1 $$ {\mathrm{Power}}\left( {{\mathrm{p}}_{{1}} } \right) = {1 }{-} \, \left( {{1}{-}p_{1} } \right)^{{\mathrm{n}}} $$

where Power(p_1_) is the power of detecting at least one infected plant for a given probability of infection (*p*_*1*_), in this case, the estimated probability (i.e. the number of infected plants in the total plants studied in the *speed main head* experiment) was used as the probability, and *n* is the number of plants evaluated. On the basis of the results obtained, a higher number of plants per treatment were studied in the *normal main head* experiment. Finally, the power was also calculated using the estimated probabilities from the *normal main head* experiment to have a better estimate and reflect the actual growing conditions recommended in this study.

### Statistical models and analyses

All the statistical analyses were performed via R software (version 4.3.1[83]). Linear mixed-effect models were built for continuous response variables (percentage of seeds not germinated, percentage of seeds not emerged, days to heading and plant height) via the lme4 package [84]. A generalized linear mixed model was built for binary response variables (head emergence and covered smut incidence) via the glmmTMB package [85]. F-tests were conducted to evaluate the effects of genotype, inoculation, and genotype-by-inoculum interactions on the response variables. The best linear unbiased estimates (BLUEs) of the response variables were estimated via the emmeans package [86]. Tukey’s pairwise multiple comparison tests were used to compare all treatments via the multicomp package [87], and contrasts tests were used to compare specific differences among treatment levels.

#### Effect of inoculum on germination, heading date, and plant height

Agronomic traits in the *speed main head* experiment, germination test, *normal main head* experiment and *normal tillering* experiment were modeled with slight modifications of the following linear mixed model:

2 $$ y_{ijkl} = \mu + G_{i} + I_{j} + \beta_{k} + GI_{ij} + \varepsilon_{ijk} + P_{l(ijk)} $$

where *y*_*ijkl*_* is* the response variable (i.e., germination, heading date or plant height), μ is the overall mean, *G*_*i*_ is the main effect of the *i*^th^ genotype, *I*_*j*_ is the main effect of the *j*^th^ inoculum level (only in *speed main head* and germination experiments), β_k_ represents the block effect, *GI*_*ij*_ is the interaction effect between the *i*^th^ genotype and the *j*^th^ inoculum level (only in *speed main head* and germination experiments), ε_ijk_ is the experimental error at the plot or experimental unit level, and *P*_*l(ijk)*_ represents the subsampling error associated with individual plant measurements when subsampling was performed (only in the *speed main head* and *normal main head* experiments). For all models, error terms were assumed to be normally distributed, such that ε_ijk_ ∼ N (0, σ^2^_e_) and *P*_*l(ijk)*_ ∼ N (0, σ^2^_s_), where σ^2^_e_ is the variance associated with the plot or experimental unit and σ^2^_s_ is the variance associated with the plant or subsampling unit_,_ with cov (ε_ijk_, *P*_*l(ijk)*_) = 0.

#### Covered smut disease incidence

Covered smut incidence and emergence were modeled for the *speed main head*, *normal main head* and *normal tillering* experiments via model (2) but following a binomial distribution. For the *normal main head* experiment, the percentage of seeds that did not emerge was also estimated via model (2).

To estimate treatment-specific odds of infection (genotype-by-inoculum or genotype), the same model structures described in (2) were implemented within a logistic regression framework. The probability of infection (R) was modeled via a logit link function, where logit(R) = log[R/(1 − R)]. The odds ratio for each treatment was then estimated in relation to the SUS check, and the results were transformed to the original units.

#### Effects of disease on head emergence, heading day, and plant height

Head emergence in the *normal main head* experiment was estimated via model (2), unconditionally for all plants of the genotypes (G_i_) and for plants of the genotypes conditional on whether they were infected (G_i_|DI = 1). Days to h eading and plant height in the *normal main head* experiment were estimated using a hurdle model approach [88], on the subset of plants which had their heads emerged to understand the difference in days to heading and plant height between plants with infection (DI = 1) and plants without infection (DI = 0) via model (3):

3 $$ y_{ijkl} = \mu + G_{i} + {\text{ D}}_{j} + {\text{ GD}}_{ij} + \, \beta_{k} + \varepsilon_{ijk} + P_{l(ijk)} $$

where *y*_*ijk*_ is the response variable head emergence of the *i*^th^ genotype, *j*^th^ disease incidence, *k*^th^ incomplete block, and *l*^th^ plant in the *normal main head* experiment in the first part of a hurdle model approach for the complete dataset; μ is the overall mean; *G*_*i*_ is the main effect of the *i*^th^ genotype; D_*j*_ is the main effect of the *j*^th^ disease incidence; GD_*ij*_ is the interaction of the *i*^th^ genotype and the *j*^th^ disease incidence; β_*k*_ is the main effect of the *k*^th^ block; ε_*ijk*_ is the experimental error at the plot level; and *P*_*l* (*ijk*)_ is the subsampling error at the plant level. Additionally, ε_*ijk*_ ∼ N (0, σ^2^_e_) and *P*_*l* (*ijk*)_ ∼ N (0, σ^2^_s_), where σ^2^_e_ is the variance associated with the plot and σ^2^_s_ is the variance associated with the plant_,_ with cov (ε_*ijk*_, *P*_*l*(*ijk*)_) = 0.

## Results

### *Speed main head* experiment

#### Disease incidence

**Fig. 2:**
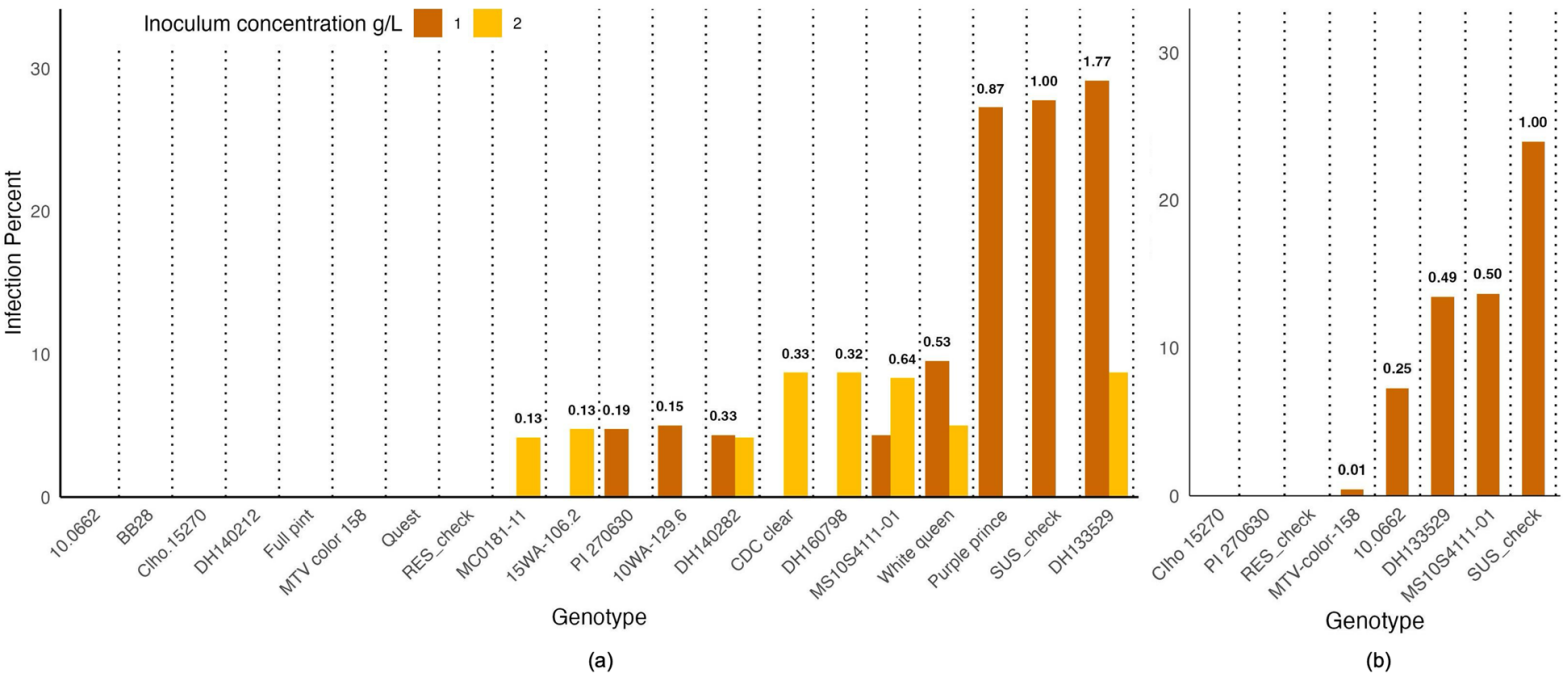
The percentage of covered smut infection is shown as bars, and the ratio of the odds of probability of infection in the genotype to the odds of probability of infection in the SUS check at 1 g L^−1^ is shown as numbers above the bars: a) *speed main head* experiment for 20 barley genotypes under two inoculum levels, 1 g L^−1^ (orange) and 2 g L^−1^ (yellow), and b) *normal main head* experiment for eight barley genotypes under one inoculum level (1 g L^−1^, orange)

Eight out of the 20 genotypes showed no disease incidence in the *speed main head* experiment (Fig. 2a). For all the genotypes infected at both inoculum levels, 1 g L^−1^ resulted in a higher infection rate than did 2 g L^−1^. Among the infected genotypes, only ‘DH133529’ at 1 g L^−1^ inoculum had higher odds of becoming infected (1.77) than did the susceptible check (SUS check at 1 g per L^−1^, odds ratio = 1.00) (Fig. 2a). The SUS check did not show infection at an inoculum concentration of 2 g L^−1^. Among the 12 infected genotypes, seven genotypes had less than a 50% chance of being infected at 1 g L^−1^ compared with the SUS check (Fig. 2a).

#### Days to heading and plant height

We had zero-inflated data with challenges in the mean and standard error (SE) estimation. Therefore, we used a hurdle model for estimating days to heading (Additional file 1: Figure S1) and plant height (Additional file 1: Figure S2) that basically subsets the dataset into genotypes with no plants infected and genotypes with at least one infected plant. For genotypes with at least one infected plant, no genotype took longer to reach heading (ZGS 59) at either inoculum level than did the non-inoculated control (Additional file 1: Figure S1b). Compared with the non-inoculated control, the SUS check was significantly shorter at both inoculum levels (ZGS 59) in genotypes in which at least one plant was infected (Additional file 1: Figure S2b). However, for the other genotypes, there was not enough power to prove that days to heading and plant height were affected by inoculation.

#### Power analysis

**Fig. 3:**
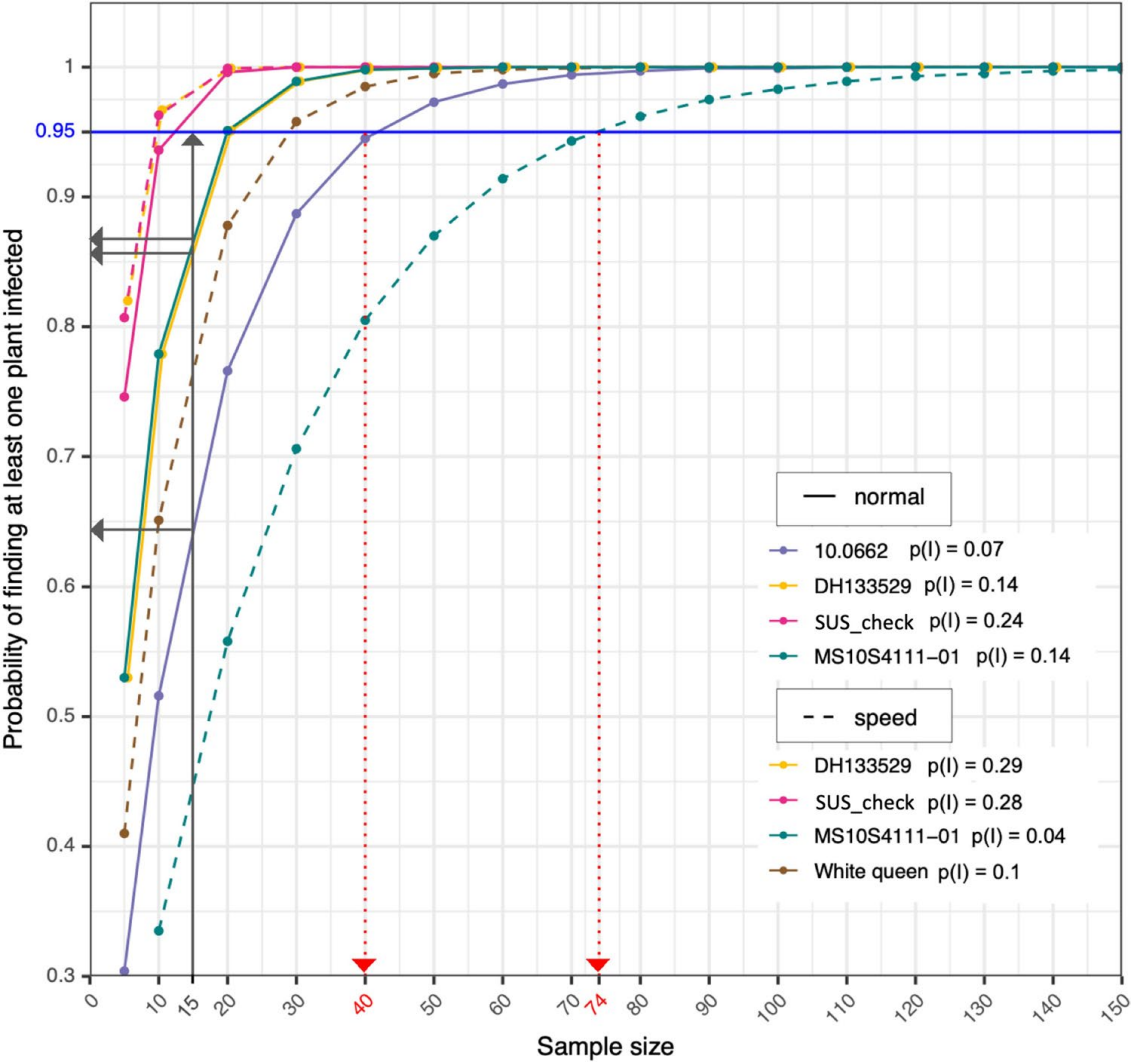
Sample size (number of plants) required to find at least one infected plant with 95% confidence for four genotypes in the *speed main head* experiment and *normal main head* experiment. The blue solid horizontal line represents the desired power (0.95). The black solid vertical line at 15 plants per line represents the sample size used by Ardiel et al. [25]. The black horizontal arrows represent the power expected for our genotypes with the Ardiel et al. [25] sample size. The red dotted vertical lines represent the sample sizes for the genotypes with the lowest infection rates in the *normal main head* (i.e. 40 plants) and *speed main head* (i.e. 74 plants) experiments. For visualization purposes, given that genotype DH133529 had the same probability of infection as MS10S4111, a jitter effect was created by adding a small value (i.e. 0.5) to the sample size of DH133529 in the figure

The minimum number of plants required to have 95% confidence in identifying at least one infected plant in the speed main head experiment was 74 for the genotypes which showed the lowest proportion of infection (Additional file 1: Table S3). No sample size was calculated for treatments with no infected plants. The power analysis comparison was done between speed main head experiment and normal main head experiment for highly and moderately susceptible genotypes (Fig. 3).

### *Normal main head* experiment

#### Effects of genotype and inoculation on disease incidence

There was a significant difference among genotypes in the probability of disease incidence in the *normal main head* experiment. Figure 2b shows a clear separation between susceptible and resistant genotypes under the new protocol. Three out of eight genotypes presented no disease incidence (Fig. 2b and Additional file 1: Table S4). The probability of infection in the SUS check (*p* = 0.239) was significantly different from all the other genotypes except for ‘MS10S4111-01’ (*p* = 0.136, Additional file 1: Table S4). ‘MTV-color-158’ had a *p* = 0.01 probability of infection in the *normal main head* experiment (Fig. 2b), but no infection was detected in the *speed main head* experiment (Fig. 2a). Additionally, MTV-color-158’ was not significantly different from the potentially resistant genotypes ‘CIho 15270’, RES check, and ‘PI 270630’ (Additional file 1: Table S4). Replicate three of ‘DH133529’ was infected with powdery mildew and were seriously damaged before reaching ZGS 59; therefore, this entry was removed from the analysis.

#### Effects of disease on head emergence, days to heading, and plant height

**Fig. 4:**
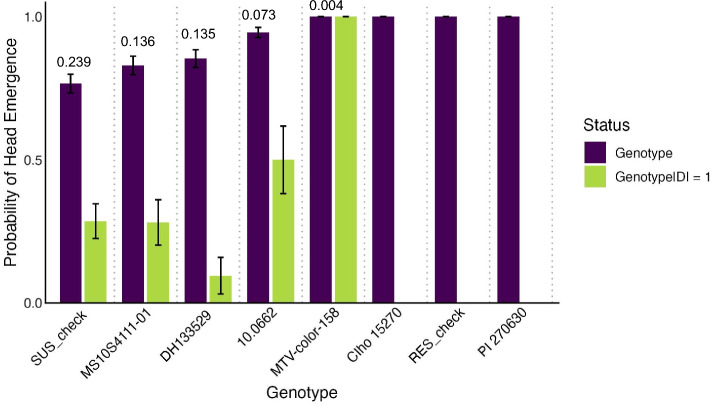
BLUEs of proportion of genotype-level head emergence is shown in dark purple (G_i_), and head emergence conditional on infection is shown in light green (G_i_|DI = 1) for eight genotypes in the *normal main head* experiment at the 1 g L^−1^ inoculum level. The lines (black) represent the standard error (SE) of each mean estimate. The numbers above bars of (G_i_) represent *p*(I) (proportion of infection for each genotype)

Head emergence was affected exclusively in diseased plants. The main heads of all potentially resistant genotypes (i.e. ‘CIho 15270’, RES check and ‘PI 270630’) emerged (Fig. 4). However, in all the susceptible genotypes except for ‘MTV-color-158’, the conditional probability of head emergence given infection (DI = 1) was lower than that in non-infected plants (Fig. 4). The genotype ‘DH133529’ presented the lowest probability of head emergence (*p* = 0.1) when infected by covered smut.

**Fig. 5:**
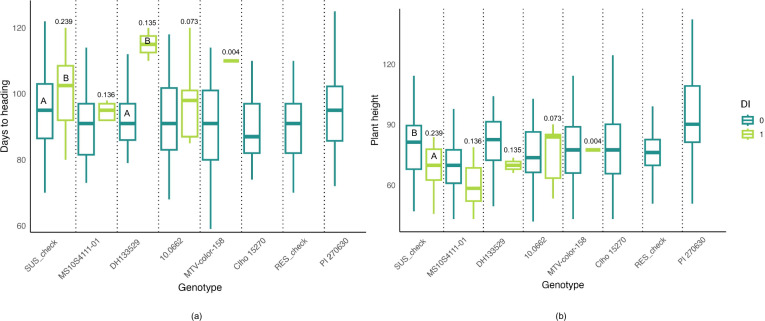
Interaction between genotype and disease incidence for plants with infection (DI = 1) (orange) and for plants without infection (DI = 0) (teal) in eight barley genotypes in the *normal main head* experiment under a 1 g L^−1^ inoculum level for **a** days to heading and **b** plant height. Genotypes that did not show any diseased plants do not have a boxplot for DI = 1 (light green). The letter comparisons show whether the means of days to heading and plant height are significantly different for plants with infection (DI = 1) and plants without infection (DI = 0) at the 5% level of significance in the contrast test. The numbers above (light green) box plots is the *p*(I) (proportion of infection for each genotype)

No significant genotype main effect was detected for days to heading in the *normal main head* experiment when comparisons were made between genotypes. However, there was a significant genotype by disease incidence interaction effect (α = 0.05) when plants with infection (DI = 1) and plants without infection (DI = 0) were compared for each genotype. In ‘DH133529’ and the SUS check, the plants with infection (DI = 1) reached heading (ZGS 59) significantly (*p* = 0.05) later than for plants without infection (DI = 0) (Fig. 5a).

On the other hand, there was a significant effect (α = 0.05) of genotype on plant height when comparisons were made between genotypes. Additionally, there was a significant interaction effect between genotype and disease incidence on plant height in the *normal main head* experiment when plants with infection (DI = 1) and plants without infection (DI = 0) were compared for each genotype (Fig. 5b). The infected plants (DI = 1) within the SUS check were shorter than non-infected plants (DI = 0) (significant at α = 0.05) (Fig. 5b).

#### Power analysis

In the power analysis conducted on the results of the *normal main head* experiment, for the genotype with moderate infection ‘MS10S4111-01’ (0.14) (Fig. 3), only 20 plants were needed to find at least one infected plant with 95% confidence. This figure represents almost one-fourth of the required number of plants for the same genotype in the *speed main head* experiment (i.e., 74 plants) (Additional file 1: Table S3, Fig. 3). With 15 plants per experimental unit, as used by Ardiel et al. [25], at least one infected plant would have been observed in genotypes ‘10.0662’, ‘DH133529’, ‘MS10S4111-01’ and ‘SUS check’, with probabilities of 0.65, 0.86, 0.86 and 0.97, respectively (Fig. 3).

### Tillering experiments

#### *Speed tillering* experiment

No covered smut infection was detected in the ‘DH133529’ genotype in the *speed tillering* experiment (Additional file 1: Figure S3). In genotype ‘SUS check’, replicates 1 and 3 did not emerge, and replicate 5 presented symptoms of covered smut incidence in all five tillers (Additional file 1: Figure S3).

#### *Normal tillering* experiment

**Fig. 6:**
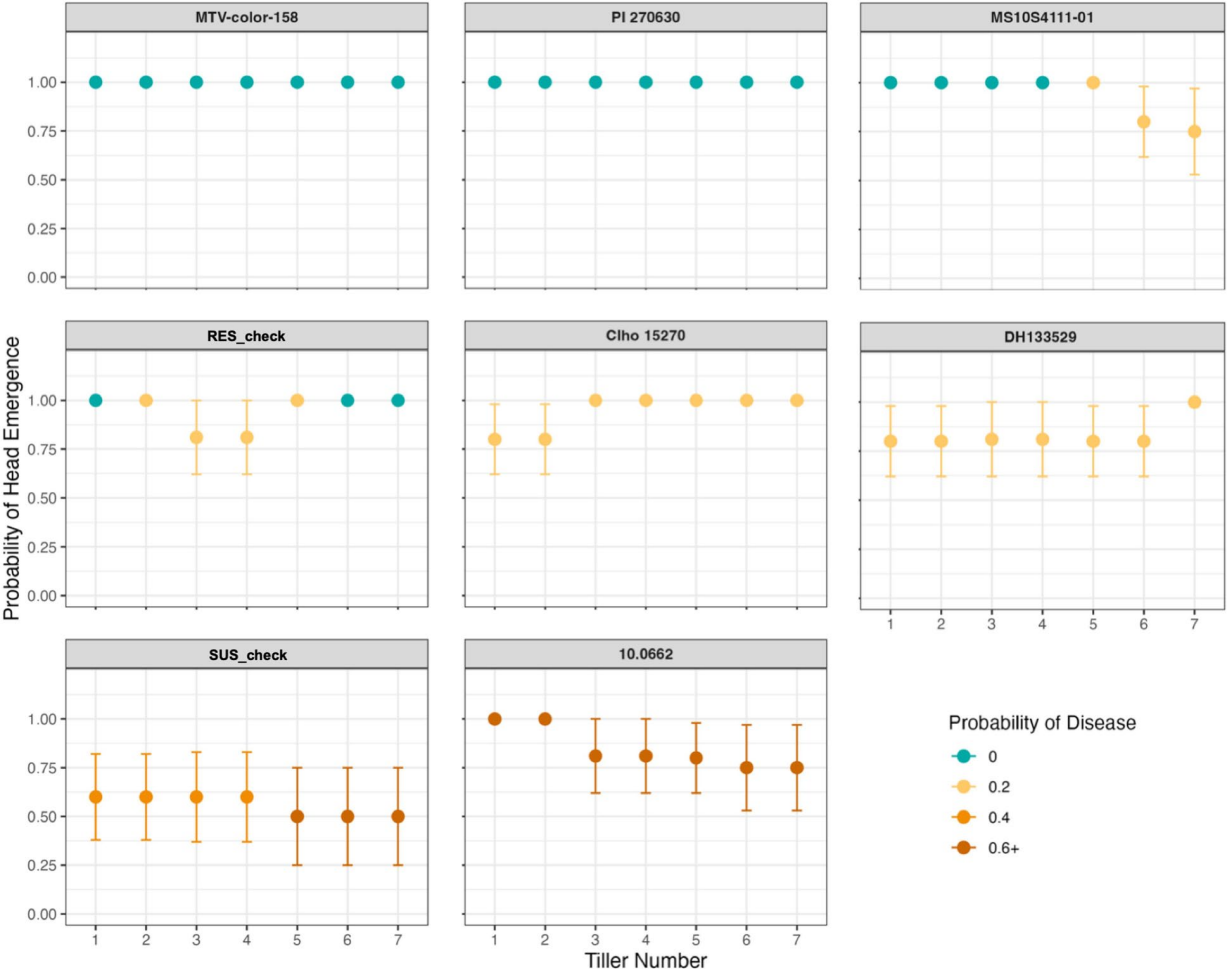
Best linear unbiased estimates (BLUEs) for the probability of head emergence in the first to seventh tillers for eight barley genotypes under the *normal tillering* experiment. The color represents the BLUEs for the probability of covered smut incidence for each tiller. The bars show the standard error (SE) of each estimate

In the *normal tillering* experiment, the genotypes ‘MTV-color-158’ and ‘PI 270630’ presented zero incidence of covered smut on the heads of any of the tillers (Fig. 6). The MS10S4111-01 genotype had an incidence of 0.4% covered smut on the heads of the latter tillers, whereas the initial tillers (1 to 4) were free of disease. In RES check, disease was observed on the heads of the second to fifth tillers; however, tillers one, six and seven were free of covered smut (Fig. 6). Genotypes ‘CIho 15270’ and ‘DH133529’ had an incidence of 0.2% covered smut on all heads, with head emergence most affected by disease incidence in genotype ‘DH133529’. For the susceptible check ‘SUS check’, heads of tillers one to four had an incidence of 0.4 of covered smut; however, those of tillers five to seven had a covered smut incidence of ≥ 0.6 (Fig. 6). This check, however, presented an overall low head emergence probability of < 0.75 for all tillers.

### Germination test

**Fig. 7:**
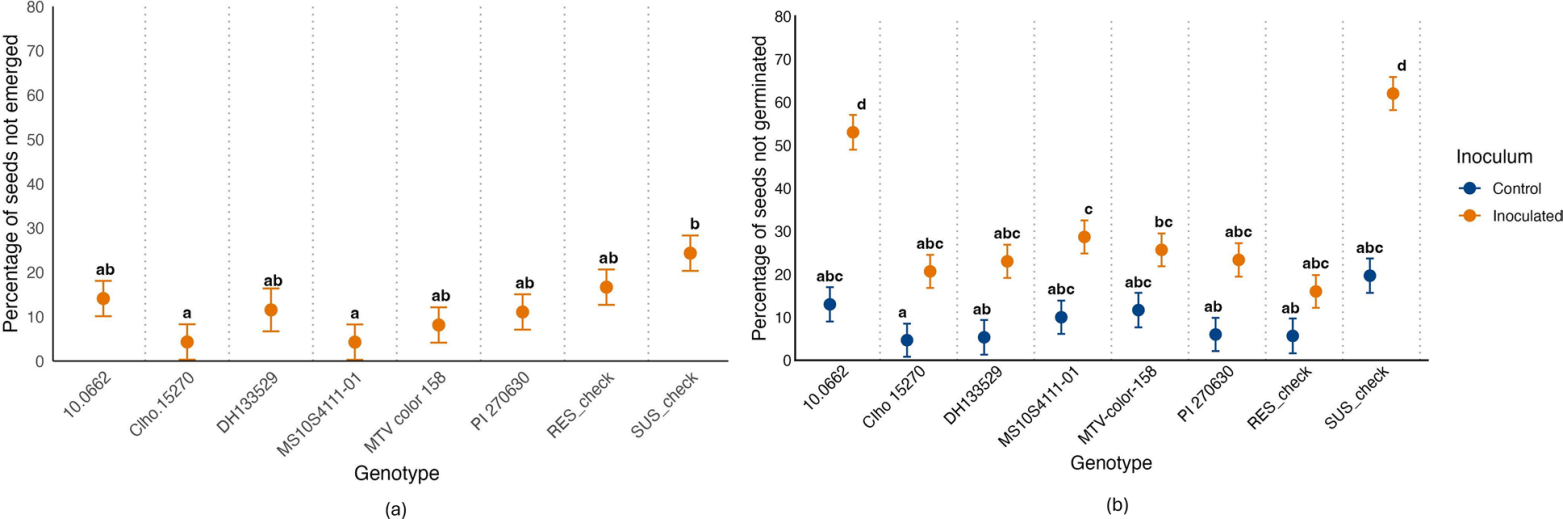
BLUEs for eight barley genotypes inoculated with covered smut under 1 g L^−1^ inoculum level for **a** the percentage of seeds not emerged in the *normal main head* experiment and **b** the percentage of seeds not germinated in the germination test. The color represents inoculated (orange) or non-inoculated control (blue) seeds. The bars show the standard error (SE) of each mean estimate. Letter comparisons show significant differences between genotypes for the traits in the *normal main head* experiment and germination tests at the 5% level of significance according to separate Tukey tests

Seed germination in the germination test was highly affected by inoculum presence (Fig. 7b), compared to seed emergence in the *normal main head* experiment (Fig. 7a and Additional file 1: Table S4). The percentage of seeds that germinated was significantly different between inoculated and non-inoculated control seeds for the genotypes SUS check and’10.0662’ in the germination test (Fig. 7b). For the SUS check, ~ 75% of the seedsemerged in the *normal main head* experiment (Fig. 7a); however, for the same genotype, 40% of the seeds germinated in the germination test (Fig. 7b).

More than 80% of the plants in the potentially resistant genotypes ‘CIho 15270’, RES check, and ‘PI 270630’ emerged well in the *normal main head* experiment (Fig. 7a and b).

## Discussion

### Covered smut incidence and seed germination

Covered smut infection is driven by multiple external factors as well as host‒pathogen interactions, making it a complex disease to study [52, 89, 90]. Disease symptoms can be observed either on the heads, where the kernels are converted to smut sori structures [7, 8, 91], or presence of fungus in the plant can be detected through PCR on leaf tissue [60]. However, PCR detection in leaves may vary depending on sampling stage, the level of susceptibility in the genotype, and is not intended to replace head-based symptom assessment [60].

For early detection of disease, we evaluated the use of a speed breeding system that would induce faster heading [70–72, 92] and therefore faster disease screening. However, the disease incidence was very low in the *speed main head* experiment for most of the infected genotypes. Even though there were significant differences in incidence between most genotypes and the susceptible check, no significant differences were detected among the non-check genotypes, possibly due to the small sample size. A lower odd of infection was observed for all the genotypes than for the SUS check, except for the genotype ‘DH133529’, which presented greater infection than did the SUS check.

In the study by Singh et al. [51], only 48% of the tested barley accessions were susceptible to smut; they claim that repeated screening is required to confirm their results. We conducted a power analysis using the results obtained in the *speed main head* experiment, which revealed that considerably more plants (i.e., 74) were needed for the least infected genotypes to observe infection in at least one plant with a 95% confidence level (Additional file 1: Table S3). Furthermore, the absence of covered smut infection in potentially susceptible genotypes could also be due to the influence of growing conditions [7, 93]. Accelerated seedling growth is observed in barley under extended photoperiods and elevated night temperatures [70], as used in speed breeding, which might have allowed some susceptible genotypes to flower faster and escape infection by outpacing fungal development.

The higher inoculum concentration (2 g L⁻^1^) resulted in lower infection rate in *speed main head* experiment. Although the causes of this remain unknown, one possible explanation is that non-linear relationships between inoculum level and infection efficiency, where excessive spore densities reduce effective infection due to factors such as spore aggregation and reduced spore germination. For example, in other pathogens, high inoculum densities form dense, slime-like masses of spores which reduce the mycelial development of fungus, indicative of altered growth dynamics, that could limit successful host colonization [94]. Therefore, only 1 g per L was used in *normal main head* experiment.

In the *normal main head* experiment (Fig. 1), where slower seedling growth was promoted with cooler conditions favorable for infection [8], we detected a significant genotypic effect on disease incidence, with the highest probability of infection in the SUS check. Genotype ‘10.0662’ (*p* = 0.073) presented a low probability of infection and may be moderately susceptible. Although higher infection rates have been reported previously using PCR on leaf tissue (Willits and Sherwood [60]), the comparatively lower infection levels observed in the susceptible check of this study is likely due to a combination of both the plant and the pathogen used, as well as the fact that we evaluated disease expression at the head. A new power analysis, conducted from the results of the *normal main head* experiment, revealed that under these conditions, a smaller sample size was still sufficient even for the genotypes with the lowest infection rates (Fig. 3). This means that the protocol used in our *normal main head* experiment was more efficient than the one used for the *speed main head* experiment, but we probably sampled more plants than needed since the conditions were better for fungal development. Finally, with the number of plants used by Ardiel et al. [25] (i.e., 15 plants), we would have been able to detect at least one infected plant with a confidence of 95% only in the most susceptible genotype SUS check. However, to observe a difference in infection between the moderately resistant genotypes, i.e., ‘10.0662’, ‘MS10S4111-01’ and ‘DH133529’, at least 40 plants are needed.

We found that some genotypes had different incidence estimates in the speed than the normal system. The lack of power in the speed breeding system could be one of the reasons for a poor estimate (i.e. the incidence estimation, and therefore the classification of genotypes into susceptible, moderately susceptible, moderately resistant, or resistance could be wrong; meaning that if the same experiment were to be repeated, different results would be obtained), but there could also be biological explanations. The normal greenhouse system, which had better pathogen growth conditions, could distinguish covered smut infection even among intermediate genotypes. Plant disease resistance is currently understood as a continuum shaped by both major (R) genes [95–97] and polygenic quantitative effects [98], with phenotypic classifications (e.g., susceptible to resistant) reflecting context-dependent outcomes influenced by pathogen population structure [99, 100], genetic background [101, 102], environment [62] and genotype-by-environment interactions [103–106]. Therefore, it is not unexpected that our systems, that tested different barley genotypes with different pathogen races in different environments, showed lower average probability of infection than previously published work. However, the fact that the normal system can find significant differences even among intermediate classes is a sign that the protocol is effective, regardless of the actual level of infection. It is also not unexpected that some genotypes changed susceptibility denomination from the speed to the normal experiments due to the level of stress plants are exposed to in the speed breeding systems.

Owing to the lower emergence rate of seedlings in the *normal main head* experiment, we decided to conduct a separate germination test to understand the effect of covered smut infection on germination. In the germination test, only two genotypes, SUS check and ‘10.0662’, presented a significantly lower percentage of seed germination when inoculated with covered smut than with the non-inoculated control; these genotypes also showed susceptibility to covered smut (Additional file 1: Table S4). Although, to our knowledge, no report of the effect of covered smut on germination has been reported, our results are consistent with previously reported germination issues due to other smut diseases by other *Ustilago* species [61, 65], as they could reduce seedling emergence by causing embryo or radicle damage [65]. Contamination of seeds by seed-borne fungi such as smuts and bunts in a range of crops can significantly compromise their storability and vigor, leading to reduced germination rates and seedling health [107, 108].

### Effects of disease on head emergence, heading, and plant height

Head emergence was negatively affected in the genotypes infected with covered smut (Fig. 4). Infected plants (DI = 1) showed a reduced head emergence when compared to all plants (unconditional of infection) of the same genotype. For the SUS check, all infected tillers presented head emergence issues. In general, biotic stress can influence flowering in barley [109] and several other studies have shown that heads affected by covered smut infection either remain enclosed within the leaf sheath or burst out prematurely, resulting in the prevention of normal heading [8, 78, 110]. Groth [9] reported that plants infected with covered smut presented delayed emergence of heads, and there was a significant difference between inoculated and non-inoculated plants. While in the *speed main head* experiment, we did not observe that covered smut infection affects days to heading, in the *normal main head* experiment, we observed that days to heading were significantly different in infected plants (DI = 1) than in uninfected plants (DI = 0). For genotypes ‘DH133529’ and SUS check, plants with infection (DI = 1) took 10–25 days longer than plants without infection (DI = 0) to reach heading. This finding agrees with studies that mention that covered smut delays heading in barley [8, 16, 78]. Some studies conducted on other spike diseases, such as common bunt in wheat [76] and pearl millet smut [77], have shown that, compared with control plants, infected plants also take longer to reach the heading stage.

We compared the plant growth of the plants at different inoculum concentrations in the *speed main head* experiment regardless of their disease expression. It is possible for a plant to be infected without expressing the disease [60] and we believe that this could still influence plant growth. The SUS check was significantly shorter in plants inoculated with 1 g L^−1^ than in non-inoculated control plants. This was also shown in the *normal main head* experiment, where there was a significant difference in plant height between plants with infection (DI = 1) and plants without infection (DI = 0) for the SUS check. Therefore, plant height was reduced due to effective infection by covered smut in barley. Nohtani et al. [79] reported that barley plants infected with covered smut presented a significant reduction in plant height. Similar disease-induced stunting has been reported in wheat, with diseases such as dwarf bunt, where infection leads to a reduced plant height and overall growth [75].

### Disease growth pattern and progression

In the *speed main head* experiment, only 75% of the infected plants presented infection of the main tiller, whereas 25% of the infected plants presented infection of secondary tillers. Therefore, we decided to assess disease across multiple tillers per plant. A difference in the probability of infection was observed among tillers within genotypes. For example, early tillers of the SUS check had a lower probability of infection (*p* = 0.4) than later tillers did (*p* = 0.6), which may be due to a greater pathogen load at the apical meristems with increasing time. In the susceptible line ‘MS10S4111-01’, covered smut was not observed in tillers 1–4 but was detected in tillers 5–7. This pattern suggests that fungal growth progresses more slowly than does host tiller development. However, beginning with the fifth tiller, infection became evident and was subsequently observed in all the later-developed tillers of ‘MS10S4111-01’. Previous histological studies have shown that fungal proliferation in barley does not occur until 42 days after inoculation, with floral meristem infection occurring approximately 54 days post-inoculation [13, 15], supporting our findings that infection escape was exhibited by the early-flowering tillers in ‘MS10S4111-01’ and in some of the early tillers of SUS check. On the other hand, Sundar et al. [111] reported that, *Ustilago* spp. may sporulate in perennial plants without visible symptoms, suggesting host tolerance under low fungal loads. In compatible interactions, disease development may occur when this tolerance is exceeded, potentially due to increased pathogen loads. A similar phenomenon has been observed in wheat blotch disease, where tolerant plants appear healthy despite pathogen infestation [112]. This could also explain why the early-flowering tillers of ‘MS10S4111-01’ were free of infection, whereas a higher mycelial load later in development may have overcome this tolerance mechanism.

Studies have shown that, in genotypes that are highly resistant to covered smut, callose deposition is observed at the initial infection site (coleoptile), which could be an effector-triggered immune response or qualitative resistance that arrests fungal growth immediately [16, 90]. However, the resistance mechanism could also be slow, similar to a PAMP-triggered response or quantitative resistance, in which fungal growth is restricted through cell wall reinforcement or phenolic deposition [11] and could be active throughout plant life. In our study, RES check showed no infection in the *normal main head* experiment or on tillers 1, 6 and 7 of the *normal tillering* experiment. It is possible that two mechanisms co-occurred in this case. First, slow fungal growth could have caused an escape of the first tiller in both experiments. Second, the plant resistance mechanism may have been activated at a later time, limiting fungal proliferation in the last few tillers, a mechanism shown by Gaudet et al. [11] and Hu et al. [13].

Genotype ‘PI 270360’ is a tall genotype that showed some infection in the *speed main head* experiment but no infection in the *normal main head* or the *normal tillering* experiments. This result contradicts the assumption that slower seedling growth under normal growing conditions favors infection. Although this could be due to estimation error, given the large sample size in the normal experiment, it is unlikely that a high power would cause a complete absence of disease in all the plants. Another possibility is that ‘PI 270360’ possesses an alternative mechanism for resistance which was deactivated under relatively high temperatures or long photoperiods, leading to infection only in the speed breeding experiment [103–106].

### Covered smut resistance screening in barley and future studies

Our SUS check revealed the highest infection percentage, similar to that reported by Grewal et al. [48], who used ‘CDC Candle’ as the susceptible check and detected differences between genotypes in terms of the probability of disease incidence. Their results confirm the effectiveness of the present protocol under normal greenhouse conditions, which are favorable for pathogen growth (Fig. 1). On the other hand, the present normal greenhouse protocol had lower infection rates than some covered smut studies, probably due to a combination of pathogen population structure [99, 100], genetic background [101, 102], environment [62] and genotype-by-environment interactions [103–106]. We believe that the present normal greenhouse protocol is highly effective because despite having a lower infection rate, it has a high precision in differentiating intermediate genotypes.

The present protocol used a greater number of plants, pH control, field soil-based growth media, and double inoculation for a better chance of infection; therefore, it could be used for screening covered smut in barley in experimental populations. The present study found that for highly susceptible lines, 10 plants would be sufficient to detect at least one infected plant, whereas for moderately resistant lines, 40 plants per experimental unit would be needed. The identification of susceptible lines in a population in the early stages of testing is beneficial for preventing the entry of susceptible material to advanced yield trials. Even though no absolute resistance was observed in RES check, ‘CIho 15270’, ‘PI 270630’ or ‘MTV-color 158’, these genotypes presented high to moderate resistance toward covered smut in the experiments we conducted. These genotypes could be used as sources to develop covered smut-resistant lines.

The development of lines with high levels of resistance in breeding populations and the use of other organically accepted treatments [44–46] could be helpful in reducing the incidence of covered smut in organic barley breeding programs and farmer fields.

## Conclusion

Artificial inoculation of covered smut in barley genotypes via different inoculum levels under a speed breeding system could not detect differences among all the genotypes for covered smut infection. The average effect of covered smut incidence on days to heading and plant height could not be observed in the speed breeding system because of possible disease avoidance/escape. Therefore, we believe that evaluating covered smut under speed breeding should be avoided. A power analysis conducted on the results of the *speed main head* experiment indicated that a greater number of plants were required to assess the disease efficiently. A modified protocol using field soil mixed with a pot mixture, pH control, and double inoculation for a relatively large number of plants in a normal greenhouse system presented significant genotypic differences and infection in some of the genotypes that were disease free in the *speed main head* experiment. The main advantage of the new protocol is that it can precisely differentiate intermediate responses. We showed that covered smut significantly impacts seed germination, head emergence, days to heading, and plant height in susceptible barley genotypes. Some genotypes exhibited delayed disease development and symptoms on fewer tillers which may be an indication of partial disease resistance. Resistant lines could be used in combination with physical and biological control in organic systems to control covered smut in barley.

## Supplementary Information

Additional file 1.

## Data Availability

The data used and/or analyzed in the current study are available through the Zenodo, which is available at Gopinathan, G. (2025). Optimization of a protocol for covered smut in barley [Dataset]. Zenodo. [47] (https:/doi.org/10.5281/zenodo.17906264).

## Abbreviations

DI: Disease incidence
ZGS: Zadok’s growth scale
BLUE: Best linear unbiased estimate
SE: Standard error
