# Supplementary material for: Covered smut screening in barley: power analysis and effect on agronomic traits
Source: Plant Methods. 2026 Jul 27;22:62. doi: 10.1186/s13007-026-01577-8 (PMC13411101; doi:10.1186/s13007-026-01577-8)
Supplement: Supplementary file 1 — Additional file 1. [file 13007_2026_1577_MOESM1_ESM.docx]

**Covered Smut Screening in Barley: power analysis and effect on agronomic traits**

Gopika Gopinathan ^1^, Fluturë Novakazi ^2^, Ines Berro ^3^, Julie Dawson ^4^, Therése Bengtsson ^5^, Lucía Gutiérrez ^5^

**Affiliations:**

^1^Department of Bacteriology, University of Wisconsin–Madison, 1550 Linden Dr., Madison, WI 53706, USA.

^2^ Department of Crop Health, Faculty of Agriculture, Civil and Environmental Engineering, University of Rostock, 18059 Rostock, Germany.

^3^ Department of Biometrics and Statistics, Agronomy College, Universidad de la República, Av. E. Garzón 780, 12900, Montevideo, Uruguay.

^4^ Department of Plant and Agroecosystem Sciences, University of Wisconsin–Madison, 1575 Linden Dr., Madison, WI 53706, USA.

^5^ Department of Plant Breeding, Swedish University of Agricultural Sciences, Sundsvägen 10, Alnarp, 23456, Sweden.

Corresponding Author: Lucía Gutiérrez, lucia.gutierrez.chacon@slu.se

**Supplementary material**

Table S1. Information on barley genotypes used in the study.

| **Genotype** | **Breeding program/location** | **Pedigree** | **Experiments** | | | | |
| --- | --- | --- | --- | --- | --- | --- | --- |
|  |  |  | ***Speed main head*** | ***Normal main head*** | ***Speed***  ***tillering*** | ***Normal***  ***tillering*** | **Germination** |
| 10.0662 | Oregon State University | KW2-849/3/Luca/Waxbar//Luca | X |  |  |  |  |
| 10WA-129.6 | Washington State University | WA 8437-95/Phoenix | X |  |  |  |  |
| 15WA-106.2 | Washington State University | X05013-T44/08WA-110.26 | X |  |  |  |  |
| BB28 | Oregon State University | Violaceum/Tamalpais | X |  |  |  |  |
| CDC Clear | University of Saskatchewan | **HB365 / CDC Trey** | X | X |  | X | X |
| CIho 15270 | USDA GRIN | - | X | X |  | X | X |
| DH133529 | Oregon State University | Alba/DZ100341 | X | X | X | X | X |
| DH140212 | Oregon State University | SH98076/10.1151 | X |  |  |  |  |
| DH140282 | Oregon State University | MC0181-11/Full Pint | X |  |  |  |  |
| RES check (DH160779) | Oregon State University | DH140030/UC1231L | X | X |  | X | X |
| DH160798 | Oregon State University | UC1266/DH140213 | X |  |  |  |  |
| SUS check (DH160799) | Oregon State University | UC1266/DH140213 | X | X | X | X | X |
| Full Pint | Oregon State University | Orca/Harrington | X |  |  |  |  |
| MC0181-11 | University of Saskatchewan | SH00752/9-1 | X |  |  |  |  |
| MS10S4111-01 | University of Minnesota | M05-29 Hu / NEG2-03 | X | X |  | X | X |
| MTV-color-158 | Oregon State University | 1_6/PI 151807 | X | X |  | X | X |
| PI270630 | USDA GRIN | - | X | X |  | X | X |
| Purple Prince | Oregon State University | Tibet violet/3/Luca/Merlin//Luca | X |  |  |  |  |
| Quest | University of Minnesota | FEG18-20/M110 | X |  |  |  |  |
| White Queen | Oregon State University | UC1266/DH140213 | X |  |  |  |  |

- pedigree information unavailable

Table S2. Method used for observation of teliospores and promycelium from smut powder and or barley seeds one day after planting.

| **Step number** | **Description** |
| --- | --- |
| Step 1 | Take 0.5 g of crushed smut powder and or seed crushed 24 hours post inoculation (1 g L^-1^ or 2 g L^-1^) and add it to a porcelain dish. |
| Step 2 | Add 10 mL of distilled water to the porcelain dish and wait for 5 minutes. |
| Step 3 | Filter excess plant material using a muslin cloth. |
| Step 4 | Add 0.5 mL of the filtered spore solution to a glass slide and observe under a compound microscope. |
| Result | 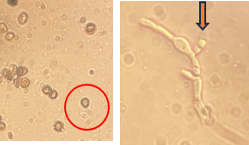 Left: Teliospore Right: Promycelium |

Figure S1. Days to heading for twenty spring barley genotypes inoculated with *Ustilago hordei* and grown under speed breeding conditions (*speed main head*) are shown in box plots for a) genotypes with no infected plants, b) genotypes with at least one infected plant. Dark blue (0) represents the non-inoculated control, orange (1) and yellow (2) the inoculum levels 1 g L^-1^ and 2 g L^-1^, respectively. The points are days to heading for the individual infected plants (DI=1). Letter comparisons show whether the mean of days to heading in non-inoculated control (dark blue) is significantly different from days to heading in 1 g L^-1^ or 2 g L^-1^ or both for a genotype at a 5% level of significance in a contrast test.

Figure S2. Plant height for twenty spring barley genotypes inoculated with *Ustilago hordei* and grown under speed breeding conditions (*speed main head*) are shown in box plots for a) genotypes with no infected plants, b) genotypes with at least one infected plant. Dark blue (0) represents the non-inoculated control, orange (1) and yellow (2) the inoculum levels 1 g L^-1^ and 2 g L^-1^, respectively. The points are plant height for the individual infected plants (DI=1). Letter comparisons show whether the mean of plant height in the non-inoculated control (dark blue) is significantly different from days to heading in 1 g L^-1^ and 2 g L^-1^ or both for a genotype at a 5% level of significance in a contrast test.

Figure S3. Raw observations for days to heading, number of tillers and covered smut-infected tillers for the two genotypes ‘DH133529’ and ‘SUS_check’ under 1 g L^-1^ inoculum used in speed-tillering experiment. Color represents Disease Incidence (DI), i.e., disease-free tillers (DI=0) (teal) and diseased tillers (DI=1) (orange). Shape represents the different replications.

Table S3. Power analysis based on the infection data in *speed main head* experiment using the cumulative distribution function to find the number of plants required per treatment (genotype-by-inoculum level) to get at least one infected plant with 95% confidence.

| **Genotype** | **Inoculum level** | | | | | | | |
| --- | --- | --- | --- | --- | --- | --- | --- | --- |
|  | **1 g L^-1^** | | | | **2 g L^-1^** | | | |
|  | **# I** | **# T** | **p (I)** | **n** | **# I** | **# T** | **p (I)** | **n** |
| **15WA-106.2** | - | 19 | - | - | 1 | 21 | 0.05 | 59 |
| **CDC clear** | - | 19 | - | - | 2 | 23 | 0.09 | 32 |
| **DH160798** | - | 14 | - | - | 2 | 23 | 0.09 | 32 |
| **MC0181-11** | - | 22 | - | - | 1 | 24 | 0.04 | 74 |
| **10WA-129.6** | 1 | 20 | 0.05 | 59 | - | 17 | - | - |
| **DH140282** | 1 | 23 | 0.04 | 74 | 1 | 24 | 0.04 | 74 |
| **MS10S4111-01** | 1 | 23 | 0.04 | 74 | 2 | 24 | 0.08 | 36 |
| **PI 270630** | 1 | 21 | 0.05 | 59 | - | 20 | - | - |
| **White Queen** | 2 | 21 | 0.10 | 29 | 1 | 20 | 0.05 | 59 |
| **SUS_check** | 5 | 18 | 0.28 | 10 | - | 15 | - | - |
| **Purple prince** | 6 | 22 | 0.27 | 10 | - | 18 | - | - |
| **DH133529** | 7 | 24 | 0.29 | 9 | 2 | 23 | 0.09 | 32 |

#I = number of infected plants, #T= total number of plants, p(I)= proportion of infection, n = number of plants required to observe at least one infected plant with 95% confidence.

Table S4. Best linear unbiased estimates (BLUEs) and mean comparisons (in parentheses) for probability of DI (Disease incidence) and No emergence (seeds did not emerge) in eight different barley genotypes in *normal main head* experiment.

| **Genotype** | **DI** | **No emergence** |
| --- | --- | --- |
| **CIho 15270** | 0.000 (a) | 0.038 (a) |
| **RES_check** | 0.000 (a) | 0.152 (ab) |
| **PI 270630** | 0.000 (a) | 0.103 (ab) |
| **MTV-color-158** | 0.004 (a) | 0.074 (ab) |
| **10.0662** | 0.073 (b) | 0.134 (ab) |
| **DH133529** | 0.135 (bc) | 0.111 (ab) |
| **MS10S4111-01** | 0.136 (cd) | 0.039 (a) |
| **SUS_check** | 0.239 (d) | 0.237 (b) |

A significant genotype effect was observed for Disease incidence (DI) and No emergence. Letter comparisons show whether the probabilities of DI (Disease incidence) and No emergence are significantly different for the genotypes at a 5% level of significance in a Tukey test.
